# A *Valsa mali* Effector Protein 1 Targets Apple (*Malus domestica*) Pathogenesis-Related 10 Protein to Promote Virulence

**DOI:** 10.3389/fpls.2021.741342

**Published:** 2021-10-07

**Authors:** Weidong Wang, Jiajun Nie, Luqiong Lv, Wan Gong, Shuaile Wang, Mingming Yang, Liangsheng Xu, Mingjun Li, Hongxia Du, Lili Huang

**Affiliations:** ^1^State Key Laboratory of Crop Stress Biology for Arid Areas, Yangling, China; ^2^College of Plant Protection, Northwest A&F University, Yangling, China; ^3^College of Horticulture, Northwest A&F University, Yangling, China

**Keywords:** defense response, *Valsa mali*, PR10, plant immunity, callose deposition

## Abstract

To successfully colonize the plants, the pathogenic microbes secrete a mass of effector proteins which manipulate host immunity. Apple valsa canker is a destructive disease caused by the weakly parasitic fungus *Valsa mali*. A previous study indicated that the *V. mali* effector protein 1 (*VmEP1*) is an essential virulence factor. However, the pathogenic mechanism of *VmEP1* in *V*. *mali* remains poorly understood. In this study, we found that the apple (*Malus domestica*) pathogenesis-related 10 proteins (*MdPR10*) are the virulence target of *VmEP1* using a yeast two-hybrid screening. By bimolecular fluorescence (BiFC) and coimmunoprecipitation (Co-IP), we confirmed that the *VmEP1* interacts with *MdPR10 in vivo*. Silencing of *MdPR10* notably enhanced the *V*. *mali* infection, and overexpression of *MdPR10* markedly reduced its infection, which corroborates its positive role in plant immunity against *V*. *mali*. Furthermore, we showed that the co-expression of *VmEP1* with *MdPR10* compromised the *MdPR10*-mediated resistance to *V*. *mali*. Taken together, our results revealed a mechanism by which a *V*. *mali* effector protein suppresses the host immune responses by interfering with the *MdPR10*-mediated resistance to *V*. *mali* during the infection.

## Introduction

The plants rely mainly on an innate complicated defense system to systematically counteract pathogen invasion. For successful infection and colonization in the hosts, the virulent pathogens deploy abundant effector proteins (EPs) which play a diverse number of roles in pathogenicity of the plant cells to modulate the plant immunity (Uhse and Djamei, 2018). The ascomycete *Valsa mali* has been reported to produce 193 secretory proteins with unknown functions, 101 of which are *V*. *mali*-specific (Li et al., 2015). These EPs are divided into two classes based on their function, inducing cell death elicitors, such as VmE02 (Nie et al., 2019) and VmHEP1 (Zhang et al., 2019), and the cell death suppressors, such as VmEP1 (Li et al., 2015) and VmPxE1 (Zhang M. et al., 2018). Among them, a deletion mutant of VmEP1 (VM1G_02400), which is a virulence factor of *V*. *mali*, showed a significantly reduction of virulence on the apple twigs and leaves (Li et al., 2015). However, how VmEP1 manipulates the host immunity is still not clear.

Several EPs have been reported to suppress the host immunity by targeting the positive regulators of immunity (Jwa and Hwang, 2017; Qi et al., 2019; Tang et al., 2020; Yang et al., 2020, 2021; Ai et al., 2021). For example, *Pseudomonas syringae* effector HopAI1 suppresses the PAMP-induced plants immunity (Zhang et al., 2007) by targeting the mitogen-activated protein kinase (MAPK), which plays roles in both the basal defense and interactions involving the R-gene-mediated resistance (Pedley and Martin, 2005). Conserved fungal effector NIS1, from *Magnaporthe oryzae*, suppresses the plants immunity by targeting PRR-associated kinases BAK1 and BIK1, which are positive regulators in the immune signaling pathway (Irieda et al., 2019; van der Burgh et al., 2019). In addition, many positive regulators targeted by the EPs have been reported. However, it remains unclear whether *V*. *mali* effector VmEP1 also targets the positive factors of immunity to suppress host immunity.

Pathogenesis-related (PR) proteins, which are key to the defense of plants, are able to enhance the disease resistance against both the biotrophic and necrotrophic phytopathogens (Honee, 1999; Jiang et al., 2015; Dai et al., 2016; Wu et al., 2016). Based on the protein sequence similarities, enzymatic activities, or other biological features, PR proteins are grouped into 17 families (Sels et al., 2008; Kim et al., 2014). Some, such as PR2, PR3, PR4, PR5, PR10, and PR12, possess significant antimicrobial activity (Loon and Strien, 1999; Chadha and Das, 2006; Sels et al., 2008; Taheri and Tarighi, 2010), and some can activate the systemic acquired resistance (SAR) pathway in the plants to defend against the phytopathogens (Ahuja et al., 2012; Navarova et al., 2012; Ali et al., 2017). Therefore, quite a few PR proteins are considered to be positive regulators of plant immunity. For this, the PR proteins can frequently be targeted by pathogen effectors. For example, the barley PR17 proteins are targeted by effector CSEP0055 from powdery mildew (Zhang et al., 2012), and wheat PR1 proteins are targeted by SnTox3 from *Parastagonospora nodorum* (Breen et al., 2016). Nonetheless, whether other PR proteins are targeted by pathogen effectors is largely unknown.

The pathogenesis-related 10 (PR10) proteins play diverse roles in the plant developmental processes, secondary metabolism, and antimicrobial activity (Choi et al., 2012). The *PR10* genes can be activated by the biotic stresses, such as microbial attacks, fungal elicitors, or wounding stresses, as well as abiotic stresses, such as salt and drought (Choi et al., 2012). In particularly, it has been shown that *PR10* from apple (*Malus domestica*) was activated when exposed to the pathogens, such as viruses and fungi (Pühringer et al., 2000; Poupard et al., 2003; Chevalier et al., 2008; Liu et al., 2021), acibenzolar-S-methyl (ASM) (Ziadil et al., 2001), ethephon, and wounding (Poupard et al., 2003), indicating that in apple, PR10 plays an important role in response to the biotic and abiotic stresses.

In this study, we showed that apple PR10 (MdPR10) was targeted by VmEP1, a virulence-essential EP from *V*. *mali* (Li et al., 2015). The transient expression of *MdPR10* in apple induced callose deposition and enhanced resistance to *V*. *mali*, whereas gene silencing of *MdPR10* in apple leaves enhanced sensitivity to *V*. *mali*, indicating that MdPR10 positively contributes to apple immunity and disease resistance, but it is unclear whether it plays a regulatory role, or directly contribute to it. We further found that the transient expression of VmEP1 compromised MdPR10-induced callose deposition and attenuated MdPR10-mediated resistance to *V*. *mali*. Our results highlight a mechanism in which a *V*. *mali* effector promoted pathogen infection by targeting a PR10 protein which positively contributes to the apple immunity.

## Materials and Methods

### Microbe and Plant Growth Conditions

The wild-type strain of *V*. *mali* 03-8 and *Sclerotinia sclerotiorum* were obtained from the Laboratory of Integrated Management of Plant Disease in the College of Plant Protection, Northwest A&F University, Shaanxi, Yangling, China (Wu et al., 2018). *Valsa mali*, *S*. *sclerotiorum*, and *Phytophthora capsici* were cultured on potato dextrose agar (PDA) at 25°C. *Agrobacterium tumefaciens* strains were routinely cultured on a yeast-extract and peptone (YEP) medium at 28°C and *Escherichia coli* strains DH5α were cultured on a Luria-Bertani medium (LB) at 37°C.

The tissue-cultured plantlets of apple (*M*. *domestica* “Gala 3”) were grown on a Murashige and Skoog (MS) agar medium containing 0.3 mg/L 6-benzyl aminopurine (6-BA) and 0.2 mg/L indoleacetic acid (IAA) (Sun et al., 2018). They were cultured under conditions of 25°C, 60 μmol/m^2^/s, and a 14 h photoperiod. *Nicotiana benthamiana* were grown in a greenhouse under a 16 h 25°C:8 h 22°C, high light intensity:darkness regime.

### Infection Assays on Leaves

*Valsa mali* and S. *sclerotiorum* were cultured on PDA for 2 days. The agar disks containing mycelium of *V*. *mali* or S. *sclerotiorum* were taken from the edge of a growing colony. The stab inoculation method was used on the infiltrated leaves of apple and *N*. *benthamiana* (Wu et al., 2018). The inoculated samples were incubated at 25°C for 36 h. The size of lesions on apple and *N*. *benthamiana* were measured by Image J software and the crossing method, respectively. The apple leaves were then collected to measure the biomass of *V*. *mali*. All the treatments were performed on at least 30 apple leaves or 10 *N*. *benthamiana* leaves, and all the experiments were repeated three times.

*Valsa mali* biomass was measured using a quantitative polymerase chain reaction (qPCR) with *V*. *mali*-specific glyceraldehyde-3-phosphate dehydrogenase (G6PDH) primers (Yin et al., 2013) related to the apple elongation factor 1 alpha gene (EF-1a) (Yin et al., 2016) at 36 h post infiltration (hpi). Genomic DNA was isolated using a Super Plant Genomic DNA Kit (Polysaccharides and Polyphenolics-rich) (Tiangen, Beijing, China) from 0.5 g apple leaves that included all the infected tissue and healthy tissue without petioles. Quantification of *V*. *mali* biomass assay was performed three times, and each experiment with three replicates.

### Plasmid Construction

All the constructs were cloned by homologous recombination with 15–20 bp of vector sequences at the 5′ terminus of each primer using a ClonExpress II One Step Cloning Kit (Vazyme Biotech, Nanjing, China). The VmEP1 (lacking the signal peptide-encoding region) was cloned from the cDNA of *V*. *mali* using gene-specific primers and was inserted into PICH86988, resulting in PICH86988-HA-VmEP1 used to perform a co-immunoprecipitation (Co-IP) assay. *MdPR10* and *NbPR10*, without terminator codons, were ligated into pCAMBIA1302 between the Nco1 and Spe1 sites, resulting in pCAMBIA1302-*MdPR10-GFP* and pCAMBIA1302-*NbPR10-GFP* for a Co-IP assay and a transient expression assay. The *VmEP1* and *MdPR10* amplified fragments were cloned into nYFP or cYFP, resulting in nYFP-MdPR10 and cYFP-VmEP1 used for carrying out a bimolecular fluorescence complementation (BiFC) assay. Individual colonies of each construct were tested by PCR and verified by sequencing. For yeast two-hybrid (Y2H) assays, VmEP1 fragment without signal peptide-encoding region was cloned into the pKGBKT7 vector using the *EcoR1* and *BamH1* sites to form the bait plasmid BD-VmEP1. MdPR10 was cloned into the pGADT7 vector using the *EcoR1* and *BamH1* sites to form the AD-MdPR10 plasmid. Loss-of-function MdPR10 plasmid was produced by cloning the reverse partially specific sequences of gene *MdPR10* into pFGC5941, carrying the *Nco*I and *Bam*HI digestion sites and resulting in pFGC5941-MdPR10 to produce siRNA (hpRNAi-MdPR10) (Zhang Q. et al., 2018). The constructs used for virus-induced gene silencing (VIGS) in *N*. *benthamiana* were generated in the tobacco rattle virus 2 (TRV2) vector (Liu et al., 2002) using the *N*. *benthamiana* cDNA library for gene fragment amplification. All the constructs were validated by sequencing in Sangon (Sangon Biotech, Shanghai, China). All the primers used above are listed in Supplementary Table 1.

### Transient Expression in *Nicotiana benthamiana* and *Malus domestica*

The constructs were transformed into *Agrobacterium* strain GV3101 (pSoup-P19) by electric shock transformation. GV3101 (pSoup-P19) containing plasmids were grown in YEP medium supplemented with appropriated antibiotics at 28°C for 16–18 h until optical density (OD)_600_ = 0.8. The bacterial cells were harvested and resuspended in the infiltration buffer (10 mM MES, 10 mM MgCl_2_, and 200 μM acetosyringone), adjusted to the required OD_600_, and incubated for 3 h before use. The plasmids were infiltrated into 4-week-old *N*. *benthamiana* leaves by injection; each plasmid was infiltrated into more than five *N*. *benthamiana* plants (∼20 leaves). For co-expression of multiple constructs, the Agrobacterium suspensions carrying the different constructs were thoroughly mixed before infiltration.

The transient expression on the apple leaves was performed as previously reported (Zhang Q. et al., 2018), with slight improvements. Agrobacterium suspensions used for transient expression in apple were used to prepare 50 ml, and the processing method was as described above. The pCAMBIA1302 constructs and pFGC5941 constructs were infiltrated into the leaves of 5-week-old tissue-cultured plantlets under a vacuum of 100 kPa for 10 min, and the infiltrated plantlets were returned to the MS medium and cultured in a growth cabinet. The infiltrated plantlets of overexpression and gene silencing were used for the experiments after 2 and 7 days of culture, respectively. Each plasmid was infiltrated into more than the eight apple plantlets (∼40 leaves).

### Yeast Two-Hybrid Assay

A Y2H system was performed to screen for VmEP1-interacting proteins (Ito et al., 2001). The cDNA library was constructed into the prey vector pGADT7 using mRNA isolated from the junction of diseased (infected by *V*. *mali* wild type strain 03-8) and healthy twigs. The recombinant BD-VmEP1 and pGADT7-cDNA libraries were co-transformed into yeast strain AH109 using the lithium acetate method according to the Yeast Protocols Handbook (Clontech, United States). To verify the interaction between VmEP1 and MdPR10, BD-VmEP1 and AD-MdPR10 were co-transformed into the yeast strain AH109. The yeast transformants were cultured on synthetic dropout medium lacking leucine, tryptothan, and medium lacking leucine, tryptophan, and histidine (SD/-Leu-Trp-His), and the positive colonies were transferred to synthetic dropout medium lacking leucine, tryptophan, and histidine and containing X-α-Gal (SD/-Leu-Trp-His + X-α-Gal) for confirmation of the interaction. The yeast transformation and interaction tests were performed following the instructions in the Yeast Protocols Handbook (Clontech, United States).

### Tobacco Rattle Virus-Based Virus-Induced Gene Silencing in *Nicotiana benthamiana*

Virus-induced gene silencing (VIGS) in plant was performed as previously described (Lu et al., 2003). A 300-bp sequence of *NbPR10*, predicted by sol genomics network (SGN) VIGS tool^1^, was amplified and cloned into pBinary TRV2 vectors between the *BamH1* and *EcoR1* sites in the antisense orientation. A TRV2 construct expressing green fluorescent protein (GFP) served as a control. The primers used for PCR amplification are listed in Supplementary Table 1. The two largest leaves of four-leaf-stage *N*. *benthamiana* plants were pressure-infiltrated with GV3101 *A*. *tumefaciens* strains containing a mixture of TRV1 (OD_600_ = 0.4) and the VIGS construct or GFP control of each target protein at OD_600_ = 0.5. The plants were used for assays or check gene-silencing levels by real-time quantitative PCR (qRT-PCR) 3 weeks later. *A*. *tumefaciens* transient expressions in combination with *P*. *capsici* infection were carried out as described previously described.

### RNA Extraction and Reverse Transcription-Quantitative Polymerase Chain Reaction

The samples were collected and ground in liquid nitrogen. For each sample, the total RNA was extracted using an EasyPure^®^ Plant RNA Kit (cat. ER301, Transgen, Beijing, China) following the instructions of manufacturer, and genomic DNA was freed by treatment with DNAse 1. A NanoDrop spectrophotometer (ND-1000, Wilmington, DE, United States) was used to quantify RNA. cDNA was synthesized using MultiScribe^TM^ Reverse Transcriptase (Thermo Fisher Scientific, MA, United States). The qRT-PCR was performed using a Roche LightCycler^®^ 96 SW1.1 Real-Time PCR System (Roche, IN, United States) with 2 × RealStar Green Power Mixture (GeneStar, Beijing, China). The EF-1a from *Malus* was used as an endogenous reference gene (Yin et al., 2016). The relative transcript level of each gene was calculated by the 2^−ΔΔ*CT*^ method (Livak and Schmittgen, 2001). All the treatments were performed in the three replicates, and all the experiments were repeated three times. The data from the three replicates were used to calculate the means and SDs. A statistical analysis was performed using Student’s *t*-test implemented by SPSS software (IBM Corp., Armonk, NY, United States) (*P* < 0.05). The PCR was performed under the following conditions: 95°C (10 min), 40 cycles at 95°C (15 s), 60°C (30 s), and 72°C (30 s), followed by a melting curve step, 95°C (15 s), 60°C (60 s), and 95°C (15 s). The gene-specific primers used for the qRT-PCR are listed in Supplementary Table 1.

The heterologous expression of *VmEP1* was verified by semi-quantitative PCR (the primers are shown in the Supplementary Table 1). cDNA products measuring 2 μl served as templates and were amplified with 2xTaq Plus MasterMix (Dye) (CWBIO, China), and in the presence of the specific primers for *VmEP1*. The gene-specific primers used for RT-PCR are listed in Supplementary Table 1. The reactions were carried out using a Life Technologies ProFlex PCR System (United States). The PCR was performed under the following conditions: 95°C (3 min); 30 cycles at 95°C (30 s), 58°C (30 s), and 72°C (30 s); 72°C (2 min); 4°C holding. The PCR products were loaded onto 1% agarose gels with Gel Green (COFIT^®^ Bioscience, China). Images of the agarose gels were acquired with a G:BOX F3 Gel Documentation System (Syngene, MD, United States).

### Immunoprecipitation

The protein fusions were overexpressed in *N*. *benthamiana* using *Agrobacterium*-mediated transient expression. The leaf samples were collected at 48 hpi. The proteins were extracted using native lysis buffer (50 mM Tris–HCl pH 7.5, 150 mM NaCl, 1 mM EDTA, 0.5% NP-40, 1 × protease inhibitor mixture) with protease inhibitor mixture and 1 mM PMSF. To immunoprecipitated GFP-tagged MdPR10 or NbPR10, the protein extracts were incubated with GFP-Trap^®^_A magnetic beads (Chromotec) for 2 h on a rotator at 4°C. The resulting samples were analyzed by immunoblotting. The samples were loaded onto a 10% sodium dodecyl sulfate–polyacrylamide gel electrophoresis (SDS-PAGE) gel run with 1 × SDS running buffer for 30 min at 10 mA and 2 h at 15 mA. The gels were blotted onto a nitrocellulose membrane for 2 h at 65 V, then stained with Ponceau solution to show loading and transfer. The membranes were blocked in 5% milk in 1 × TBST [TBS (50 mM Tris–HCl, 150 mM NaCl, and pH 7.4) with Tween-20 0.1% (vol/vol)] for 1 h before the addition of primary antibodies overnight: either a monoclonal GFP antibody raised in mouse at 1:1,000 dilution (cat. AF0159; Beyotime) or a monoclonal hemagglutinin (HA) antibody raised in mouse at 1:1,000 (cat. no. 26183; Abcam). The membrane was washed with 1 × TBST (0.1% Tween 20) three times for 10 min each before the addition of the secondary antibody at 1:8,000 dilution with goat anti-mouse lgG-HRP antibody (cat. A0216; Beyotime) for 1 h. ECL Western Blotting Substrate (cat. PE0030; Beijing Solarbio Science and Technology, Beijing, China) was used according to the instructions of manufacturer.

### Callose Staining

Callose deposition of *N*. *benthamiana* and apple leaves were stained with aniline blue as described (Adam and Somerville, 1996) and observed using a fluorescence microscope. Callose accumulation was quantified using Image J software and data were analyzed by Student’s *t*-test or Tukey’s multiple comparisons test.

### Confocal Fluorescence Microscopy

The patches of *N*. *benthamiana* leaves were imaged 48 h after agro-infiltration using an Olympus FV3000 laser scanning microscope (Olympus Corporation, Tokyo, Japan). The GFP and yellow fluorescent protein (YFP) were imaged at excitation/emission wavelengths of 488 and 561 nm, respectively.

## Results

### Transient Expression of a *Valsa mali* Effector Protein 1 in Plant Enhances Susceptibility to Pathogens

**FIGURE 1:**
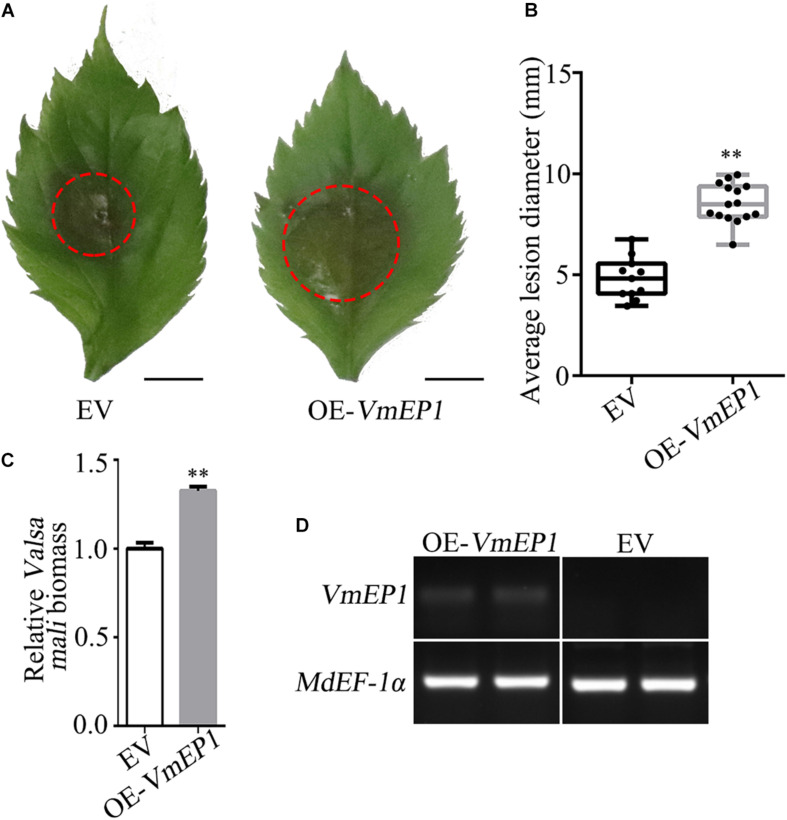
The transient expression of *Valsa mali* effector protein 1 (*VmEP1*) in apple facilitates *V*. *mali* infection. **(A)** Representative disease symptoms of the apple leave overexpressing *VmEP1* at 36 hpi of *V*. *mali*. Bars = 5 mm. **(B)** The average lesion diameter of apple leaves in which *VmEP1* is overexpression was evaluated at 36 hpi of *V*. *mali*. **(C)** *V*. *mali* biomass was measured with qPCR at 36 hpi of *V*. *mali*. The relative *V*. *mali* biomasses were normalized to the mean of the wild-type. **(D)** A semiquantitative RT-PCR analysis to assess the expression of *VmEP1*. **(A–D)** EV, pCAMBIA1302 empty vector; OE-*VmEP1*, apple leaves overexpressing *VmEP1*. Mean ± SD; *n* > 10; ^∗∗^*P* < 0.01; Student’s *t*-test.

The VmEP1 was previously demonstrated to contribute to *V*. *mali* full virulence (Li et al., 2015). As an approach to illustrate this, VmEP1 lacking the signal peptide was transiently expressed in the apple leaves followed by inoculation with *V*. *mali*. As a result, the average number of lesions in the leaves expressing *VmEP1* increased by ∼49% when compared with those expressing GFP control (Figures 1A,B). The relative biomass of *V*. *mali* in the apple leaves expressing *VmEP1* was about 1.3 times that of the control (Figure 1C). The expression of *VmEP1* was determined by semiquantitative RT-PCR (Figure 1D). These results indicated that the transient expression of *VmEP1* in the apple leaves enhanced the host susceptibility to *V*. *mali*.

We also tested whether VmEP1 could facilitate the infection of other phytopathogens. For this, the VmEP1 was transiently expressed in the model plant *N*. *benthamiana* prior to inoculation with *P*. *capsici* and *S*. *sclerotiorum*. As shown (Supplementary Figures 1A–D), the average lesion diameters caused by *P*. *capsica* and *S*. *sclerotiorum* in the leaves expressing VmEP1 were ∼19 and ∼14% higher than those expressing the GFP control, respectively. These results indicated that VmEP1 could enhance the plant susceptibility to both oomycetes (*P*. *capsici*) and fungi (*V*. *mali* and *S*. *sclerotiorum*).

### A *Valsa mali* Effector Protein 1 Interacts With Apple (*Malus domestica*) Pathogenesis-Related Protein 10

**FIGURE 2:**
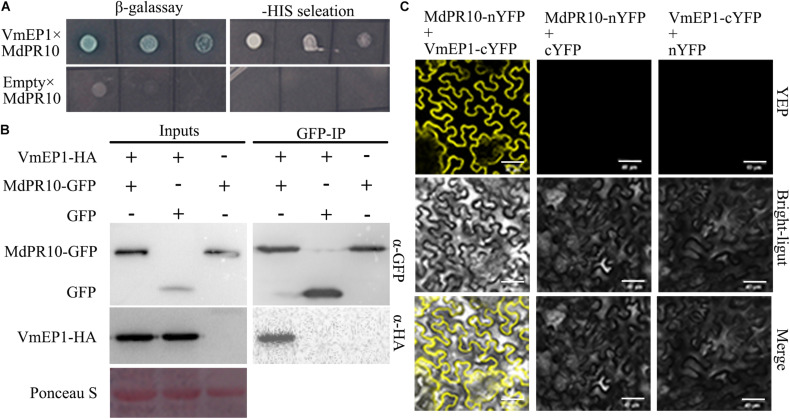
Interaction between *VmEP1* and *Malus domestica* pathogenesis-related 10 protein (*MdPR10*) *in vitro* and *vivo*. **(A)** The positive yeast clones were cultured on SD-4 + X-α-Gal with ladder concentration of yeast suspension (10^–1^, 10^–2^, and 10^–3^) for further confirmation. **(B)** Bimolecular fluorescence complementation showed that *VmEP1* interacted with *MdPR10* in the leaf cells of *N*. *benthamiana*. VmEP1-cYFP and MdPR10-nYFP were co-expressed in *N*. *benthamiana* by agroinfiltration. The yellow fluorescence was observed 48–72 h post infiltration (Bars = 20 μm). **(C)** *In vivo* Co-IP assay of HA: *VmEP1* (without the signal peptide sequence) and green fluorescent protein (GFP)-*MdPR10*. Both the genes were expressed in *N*. *benthamiana* leaves using agro-infection. The input experiment was performed by western blot with the HA antibody and GFP antibody to confirm the expression of the two proteins. The mixed proteins were blended with GFP-trap agarose beads. The final eluent was analyzed by immunoblot using the above-mentioned antibodies to detect *VmEP1* and *MdPR10*. This assay was repeated three times.

To clarify the underlying virulence mechanism of VmEP1, we aimed to find those host components that it targets. For this, we adopted the Y2H assay approach to screen the candidate targets using VmEP1 as bait. MdPR10 was one of the candidate proteins screened by Y2H assay (Figure 2A). To validate the VmEP1-MdPR10 interaction, a reciprocal BiFC assay was first performed. It showed that VmEP1 interacted with MdPR10 *in vivo* (Figure 2). To further confirm their interaction, we performed a Co-IP assay in *N*. *benthamiana*. As shown in Figure 2C, a specific signal for VmEP1-HA was clearly observed in the MdPR10-GFP immunoprecipitated (Figure 2C), indicating that VmEP1 interacted with MdPR10 in the plants.

Since VmEP1 also facilitates pathogen infection in *N*. *benthamiana*, we analyzed the homologous sequence of MdPR10 in *N*. *benthamiana* (Supplementary Figure 2A) and tested whether it interacted with VmEP1 similarly. As expected, the BiFC and Co-IP assays showed that the yellow fluorescence and a specific signal for VmEP1 were observed (Supplementary Figures 2B,C), indicating that the VmEP1 also interacted with *N*. *benthamiana* PR10 (NbPR10). In addition, we tested the interaction between an empty vector cYFP and nYFP. The yellow fluorescence was not detected (Supplementary Figure 3), indicating the results observed were all positive results.

### Apple (*Malus domestica*) Pathogenesis-Related Protein 10 Positively Contributes to Plant Immunity

**FIGURE 3:**
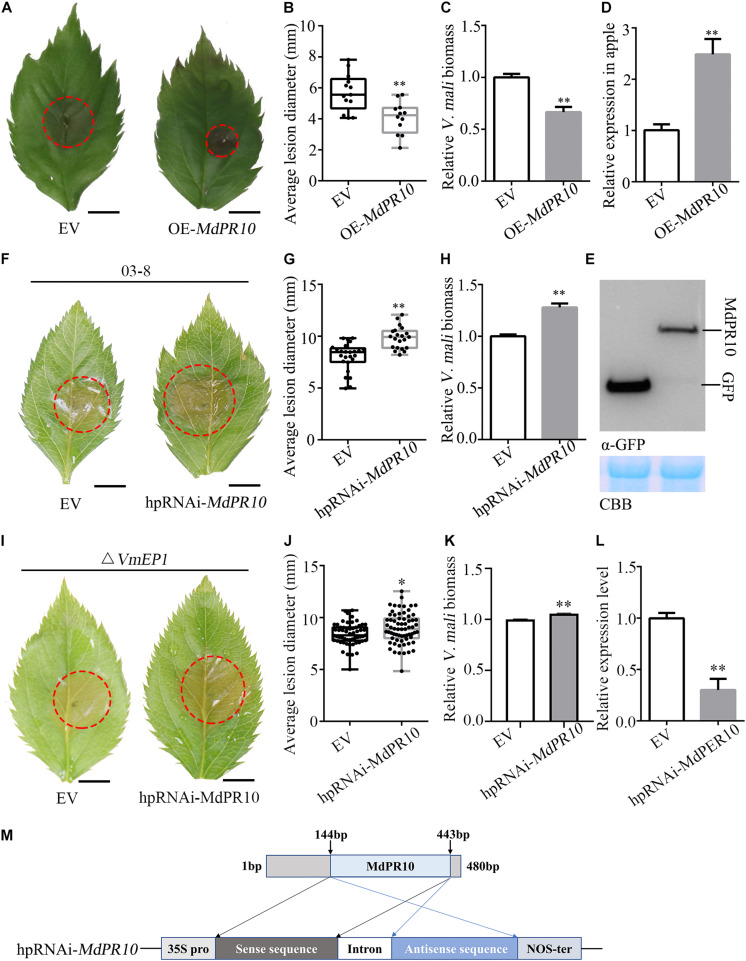
Apple (*Malus domestica*) pathogenesis-related protein 10 positively contributes apple resistance to *V*. *mali*. **(A)** Representative disease symptoms of the apple leaves transiently expressing *MdPR10* at 36 h after inoculation of *V*. *mali*. Bars = 5 mm. **(B)** The average lesion diameter in the apple leaves in which MdPR10 is transiently expressed was evaluated at 36 hpi of *V*. *mali*. **(C)** The mRNA level of *MdPR10* in the apple leaves, as revealed by reverse transcription-quantitative polymerase chain reaction (RT-qPCR) 2 days after infiltration of OE-*MdPR10*. **(D)** *V*. *mali* biomass was measured with qPCR at 36 hpi. **(A–D)** EV, pCAMBIA1302 empty vector; OE-*MdPR10*, the apple leaves overexpressing *MdPR10*. **(E)** Immunoblot analysis of proteins in apple transiently expressing GFP control and MdPR10 fused with GFP tag. **(F)** Representative disease symptoms of the apple leaves in which *MdPR10* is silenced at 36 hpi of *V*. *mali*. Bars = 5 mm. **(G)** The average lesion diameter in the apple leaves in which MdPR10 is silenced was evaluated at 36 hpi of *V*. *mali*. **(H)** *V*. *mali* biomass was measured with qPCR at 36 hpi of *V*. *mali*. Relative *V*. *mali* biomasses were normalized to the mean of the wild-type. **(I)** Representative disease symptoms of the apple leaves silencing *MdPR10* after inoculation of *VmEP1* deletion mutants of *V*. *mali*. Bars = 5 mm. **(J)** The average lesion diameter in the apple leaves in which MdPR10 is silenced was evaluated at 36 h after the inoculation of *VmEP1* deletion mutants of *V*. *mali*. **(K)** The *V*. *mali* biomass was measured with qPCR at 36 hpi of the *VmEP1* deletion mutants of *V*. *mali*. The relative *V*. *mali* biomasses were normalized to the mean of the wild-type. **(L)** Silence efficiency detection of MdPR10 in apple leaves by RT-qPCR 5 days after the infiltration of hpRNAi-*MdPR10*. **(M)** Schematic of the constructs used to silence MdPR10. hpRNAi-*MdPR10* were cloned into the pFGC5941 binary vector. In panels **(E–L)** EV, pFGC5941 empty vector; hpRNAi-*MdPR10*, apple leaves in which *MdPR10* is silenced. In panels **(B,F,J)** mean ± SD; *n* > 10; ^∗^*P* < 0.05, ^∗∗^*P* < 0.01; Student’s *t*-test. In panels **(C,D,G,H)** mean ± SD; *n* = 3; ^∗∗^*P* < 0.01; Student’s *t*-test. The genome DNA of the apple leaves was extracted and the relative *V*. *mali* biomass was estimated by DNA-based quantitative PCR (qPCR). These experiments were repeated three times with similar results.

To investigate the role of MdPR10 during phytopathogen infection, we infiltrated *A*. *tumefaciens* cells carrying an MdPR10 fusion construct in the apple leaves, followed by inoculation with *V*. *mali*. The average lesion diameter of apple leaves expressing MdPR10 was ∼43% smaller than that of the control (Figures 3A,B). Moreover, the relative biomass of *V*. *mali* in the apple leaves expressing MdPR10 was ∼0.6 times that of the control (Figure 3C). The qRT-PCR results showed that the gene expression level of MdPR10 increased significantly (Figure 3D) and immunoblot analysis of MdPR10-GFP indicated MdPR10 was successfully expressed (Figure 3E). We also inoculated the apple leaves with *Colletotrichum gloeosporioides*. The results showed that the lesion diameter on the apple leaves expressing MdPR10 decreased (Supplementary Figure 4). To expound on these results, we further silenced MdPR10 in the apple leaves by *Agrobacterium*-mediated transient expression and inoculated the leaves with *V*. *mali*. The average lesion diameter of the apple leaves silencing MdPR10 was ∼25% higher than that of the control (Figures 3F,G). The relative biomass of *V*. *mali* in the apple leaves silencing MdPR10 was ∼1.27 times that of the control (Figure 3H). The qRT-PCR analysis showed that the transcript levels of MdPR10 were reduced by ∼50% (Figure 3L). Figure 3M illustrates a schematic diagram of the construction of the MdPR10 silencing vector. These results collectively suggested that MdPR10 positively contributed to the apple immunity and disease resistance.

To further confirm the above results, we transiently expressed *MdPR10* in *N*. *benthamiana* and inoculated with *P*. *capsici*. The average lesion diameter of *N*. *benthamiana* leaves expressing *MdPR10* was ∼32% smaller than that of the control (Supplementary Figures 5A,B). Furthermore, we silenced *NbPR10* in *N*. *benthamiana* by VIGS and inoculated *P*. *capsici*. It showed that the average lesion diameter of *N*. *benthamiana* leaves silencing *NbPR10* was ∼29% larger than that of the control (Supplementary Figures 5C,D). The silencing efficiency of *NbPR10* was tested by qRT-PCR, and the result showed that the *NbPR10* expression level was reduced by ∼80% (Supplementary Figure 4E). These results indicated that NbPR10 positively contributed to the plant immunity and disease resistance as well.

To address if the PR10 is a virulence target of VmEP1, *VmEP1* deletion mutants (Li et al., 2015) were inoculated on the apple leaves silencing *MdPR10*. The average lesion diameter of the *VmEP1* deletion mutants was ∼14% higher than that of the control (Figures 3I,J). The relative biomass of *V*. *mali* in the apple leaves silencing MdPR10 was significantly greater than that of the control (Figure 3L). These results suggested that PR10 was a functional target for VmEP1. The relative lesion diameter growth rate of *V*. *mali* (∼25%) was higher than that of the *VmEP1* deletion mutants (∼15%), indicating PR10 was essential for VmEP1 virulence function. These results indicated that the PR10 was a functional target for VmEP1.

### A *Valsa mali* Effector Protein 1 Suppresses Apple (*Malus domestica*) Pathogenesis-Related 10 Proteins- and Pathogen-Associated Molecular Pattern-Triggered Callose Deposition

**FIGURE 4:**
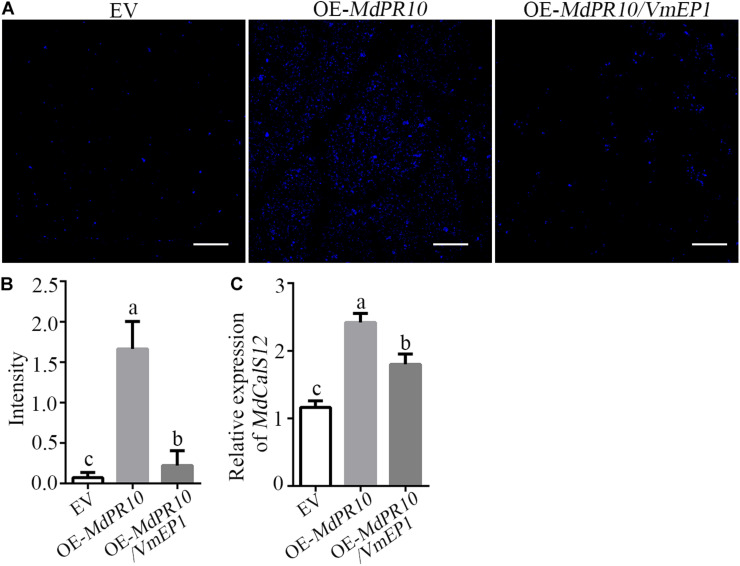
A *Valsa mali* effector protein 1 suppresses *MdPR10*-mediated callose deposition. **(A)** Aniline blue staining with leaves at 48 h after expression of *MdPR10* or co-expression of *MdPR10* and *VmEP1* in the apple leaves. Bars = 100 μm. The expression of EV as control. Bars = 50 μm. **(B)** Quantification of the callose deposition in apple leaf tissues, as determined by ImageJ software. Mean ± SD; n > 10; a, b, and c indicate *t-test P* < 0.05; Duncan’s multiple range test. **(C)** The mRNA level of *MdCalS5* in the apple plantlets, as revealed by qRT-PCR 2 days after infiltration of OE-*MdPR10*. The transcript levels were analyzed by qRT-PCR and normalized to that in the EV using the EF-1α gene as an internal reference. Mean ± SD; *n* = 3; a, b, and c indicate *t-test P* < 0.05; Duncan’s multiple range test. These experiments were repeated three times with similar results. Panels **(A–C)** EV, pCAMBIA1302 empty vector.

We detected the callose deposition by staining with aniline blue after transient expression of *MdPR10*. The results showed that the accumulation of callose was much greater in the apple leaves expressing *MdPR10* than that of the empty vector (Figure 4). However, the callose deposition in the apple leaves co-expressing *MdPR10* and *VmEP1* was clearly attenuated, compared with the experimental group expressing *MdPR10* alone (Figure 4). To validate these results, we detected the transcript level of *MdCalS5*, a key enzyme for callose synthesis (Xie et al., 2012), by qRT-PCR. It showed that the expression level of *MdCalS5* in the apple leaves expressing *MdPR10* was about two times higher than the control expressing the empty vector alone. Additionally, the co-expression of MdPR10 and VmEP1 attenuated the transcript level of *MdCalS5* by ∼34% compared with the apple leaves expressing *MdPR10* (Figure 4C). These results indicated MdPR10 triggered callose deposition, and VmEP1 inhibited the response mediated by MdPR10.

To verify whether the expression of VmEP1 affected the pathogen-associated molecular pattern (PAMP)-triggered callose accumulation, we examined the callose deposition in *N*. *benthamiana* leaves after treatment with INF1 elicitin (Kamoun et al., 1997), a PAMP of *Phytophthora infestans* (Kamoun et al., 1998). The result showed that VmEP1 significantly suppressed the callose deposition induced by INF1 (Supplementary Figure 6). Together, these results indicated that VmEP1 could effectively inhibit the accumulation of callose in the plants.

### A *Valsa mali* Effector Protein 1 Compromises Apple (*Malus domestica*) Pathogenesis-Related 10 Proteins-Mediated Resistance

**FIGURE 5:**
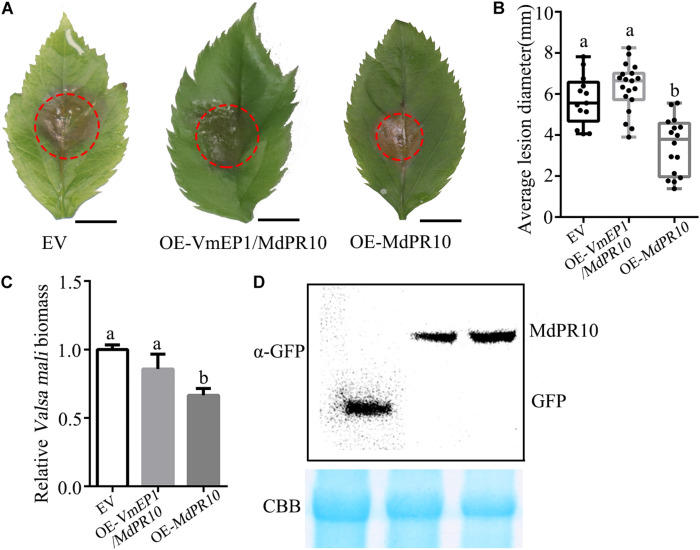
A *Valsa mali* effector protein 1 attenuates the MdPR10-mediated resistance to *V*. *mali*. **(A)** Representative disease symptoms of the apple leaves overexpressing the genes at 36 hpi of *V*. *mali*. Bars = 5 mm. **(B)** The average lesion diameter in the apple leaves in which genes overexpression was evaluated at 36 hpi of *V*. *mali*. Means ± SDs; *n* > 15; a and b indicate *t-test P* < 0.05; Duncan’s multiple range test. **(C)** *V*. *mali* biomass was measured with qPCR at 36 hpi of *V*. *mali*. The relative *V*. *mali* biomasses were normalized to the mean of the control. Means ± SDs; *n* = 3; a and b indicate *t-test P* < 0.05; Duncan’s multiple range test. The genome DNA of apple leaves was extracted and the relative *V*. *mali* biomass was estimated by DNA-based quantitative PCR (qPCR). These experiments were repeated three times with similar results. **(D)** Immunoblot analysis of the expressed proteins were performed with anti-GFP antibody. CBB staining (bottom) was used as a loading control.

Since VmEP1 attenuated the accumulation of callose induced by MdPR10, we speculated that VmEP1 might affect the resistance of MdPR10 to phytopathogens. To test this hypothesis, MdPR10 and VmEP1 were transiently co-expressed in apple. A *V*. *mali* inoculation assay showed that the average lesion diameter of the apple leaves co-expressing VmEP1 and MdPR10 was similar with that of the control, and was about 43% higher than the average lesion diameter of the apple leaves expressing MdPR10 (Figures 5A,B). These results indicated that the function of MdPR10 to suppress the infection of *V*. *mali* was weakened by VmEP1. The changed trend of relative *V*. *mali* biomass was consistent with the average lesion diameter (Figure 5C). Immunoblot analysis showed that MdPR10 successfully expressed (Figure 5D). These results showed that VmEP1 promoted infection of phytopathogens by inhibiting the callose deposition induced by MdPR10.

## Discussion

To resist the biotic and abiotic stress, the plants initiate a series of immune responses, such as reactive oxygen species (ROS) bursts, biosynthesis of phytohormones, the expression of a large number of defense-related genes, and callose deposition. The callose deposition at the cell wall is an early defense response (Jones and Dangl, 2006; Schwessinger and Ronald, 2012), which is associated with cell death (Han and Hwang, 2017). In *Arabidopsis*, the measuring callose deposition has become a popular model system to quantify the plant immunity activity (Luna et al., 2011), since pathogen invasion could be slowed by the cell walls thickened by the deposition of callose (Nishimura et al., 2003).

From the previous studies, we knew that the overexpression of *CaPR10* and *PpPR10* can induce the callose deposition (Choi et al., 2012; Castro et al., 2016), PR10 of *Malus sieversii* is upregulated after infection with a 15-fold higher increase than the control (Liu et al., 2021), and the transient expression of PR10 enhances the resistance to phytopathogens, such as PR-10 from *Panax ginseng* C. A. Meyer (Pulla et al., 2010), *Zea mays* (Xie et al., 2010), and strawberry (*Fragaria x ananassa*) (Besbes et al., 2019). This means the PR10 proteins, one of the pathogenesis-related groups, may contribute to plant resistance by inducing the callose deposition. In this study, we found that the overexpression of *MdPR10* induced callose deposition in the apple leaves (Figure 4) and enhanced the resistance of apple leaves to *V*. *mali* (Figures 3A–C). Our experimental results confirmed the previous studies, but the mechanism of immune activation of PR10 is still unclear.

Conversely, to successfully invade and colonize their host plants, the phytopathogenic bacteria, fungi, and oomycetes can secret diverse groups of EPs to inhibit the callose deposition, such as PsCRN63 from *Phytophthora sojae* (Li et al., 2016), PSTha5a23 from *Puccinia striiformis* f. sp. tritici (Cheng et al., 2017), and Cce1 from *Ustilago maydis* (Seitner et al., 2018). In our study, we found that the EP VmEP1 could not only inhibit the accumulation of callose (Figure 4 and Supplementary Figure 6), but also promote the infection of *V*. *mali* (Figure 1 and Supplementary Figure 1) by interaction with MdPR10 (Figure 2). The phytophthora effectors can affect the various aspects of the host plant immune systems to manipulate the host immunity, such as plant cell proteases, phytohormones, RNAs, the MAPK pathway, catalase, the ubiquitin proteasome pathway, the endoplasmic reticulum, nucleotide binding leucine-rich repeat (NB-LRR) proteins, and the cell membrane (Wang and Jiao, 2019). It has also been reported that PR10 interacting with effector CSEP0064/BEC1054, secreted by the fungal pathogen *Blumeria graminis*, may modulate the antimicrobial activity of these defense-related polypeptides (Dodds et al., 2019). Hence, we speculate that VmEP1 disturbs the host plant immune systems by interacting and interfering with MdPR10. However, it is still unknown how the effectors manipulate the function of PR10 proteins to interfere in the host plant immune response. Interestingly, the overexpression of *PR10* can activate a plant defense response, which is increased by its interaction with leucine-rich repeat (LRR1) proteins (Choi et al., 2012). Therefore, we put forward a hypothesis that VmEP1 and LRR1 may competitively interact with PR10, resulting in the weakening or disappearance of the interaction between LRR1 and PR10. Of course, this hypothesis needs to be verified by further research.

In summary, this study helps illustrate the mechanism of how VmEP1 aids infection of *V*. *mali*. MdPR10 was identified as a target of VmEP1 and a mediator of the defense response of the plants to inhibit the infection of phytopathogens. For successful colonization, *V*. *mali* secretes effector VmEP1 to disturb MdPR10 resistance to *V*. *mali*. The discovery of MdPR10-enhanced resistance to *V*. *mali* will provide a new guidance for breeders to engineer the disease-resistant plants. Further studies are needed to explain how VmEP1 interferes in the function of MdPR10.

## Data Availability Statement

The original contributions presented in the study are included in the article/Supplementary Material, further inquiries can be directed to the corresponding author.

## Author Contributions

WW and LH designed the research. WW mainly contributed to the all experiments. WG, LL, SW, and HD assisted with specific experiments. JN, MY, LX, and ML assisted with preparation of the manuscript. LH revised the manuscript. All authors contributed to the article and approved the submitted version.

## Conflict of Interest

The authors declare that the research was conducted in the absence of any commercial or financial relationships that could be construed as a potential conflict of interest.

## Publisher’s Note

All claims expressed in this article are solely those of the authors and do not necessarily represent those of their affiliated organizations, or those of the publisher, the editors and the reviewers. Any product that may be evaluated in this article, or claim that may be made by its manufacturer, is not guaranteed or endorsed by the publisher.

## Footnotes

1
https://vigs.solgenomics.net/
