# Supplementary material for: A Valsa mali Effector Protein 1 Targets Apple (Malus domestica) Pathogenesis-Related 10 Protein to Promote Virulence
Source: Front Plant Sci. 2021 Oct 7;12:741342. doi: 10.3389/fpls.2021.741342 (PMC8528966; doi:10.3389/fpls.2021.741342)
Supplement: Supplementary file 1 [file Data_Sheet_1.docx]

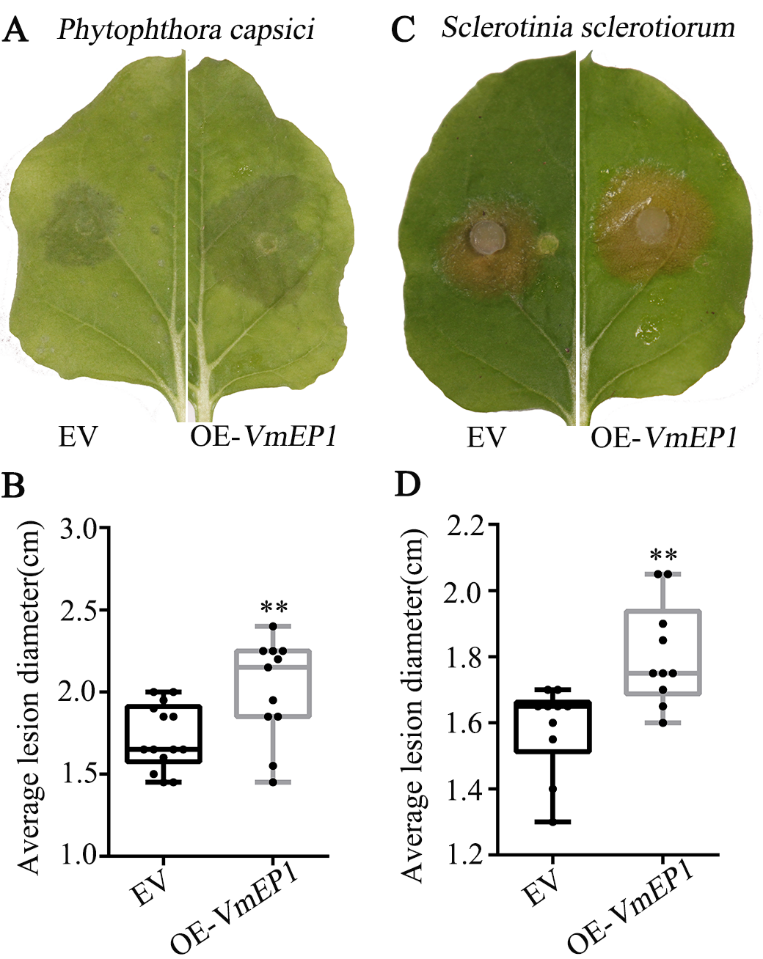


**Supplementary Figure 1.** (A) Representative disease symptoms of EV- or *VmEP1*-overexpressed *N. benthamiana* infected with *P. capsica*. The leaves (n>10) were inoculated with *P. capsica* fresh mycelial plugs and photographed 36 h post-inoculation (hpi). The experiment was repeated three times with similar results. (B) The lesion diameter was evaluated 36 h after inoculation of *P. capsica*. (C) Representative disease symptoms of EV- or VmEP1-overexpressed *N. benthamiana* infected with *S. sclerotiorum*. The leaves (n>10) were inoculated with *P. capsica* fresh mycelial plugs and photographed 36 h post-inoculation (hpi). The experiment was repeated three times with similar results. (D) The lesion diameter was evaluated 36 h after inoculation of *S. sclerotiorum*. (E) Semiquantitative RT-PCR analysis to assess the expression of VmEP1. (A) to (E): EV, pCAMBIA1302 empty vector; OE-*VmEP1*, transient expression of VmEP1 in *N. benthamiana*. In (B) and (D):Error bars represent standard deviations calculated from three biological replicates. The statistical analyses were performed with Student’s t test: **P<0.01.


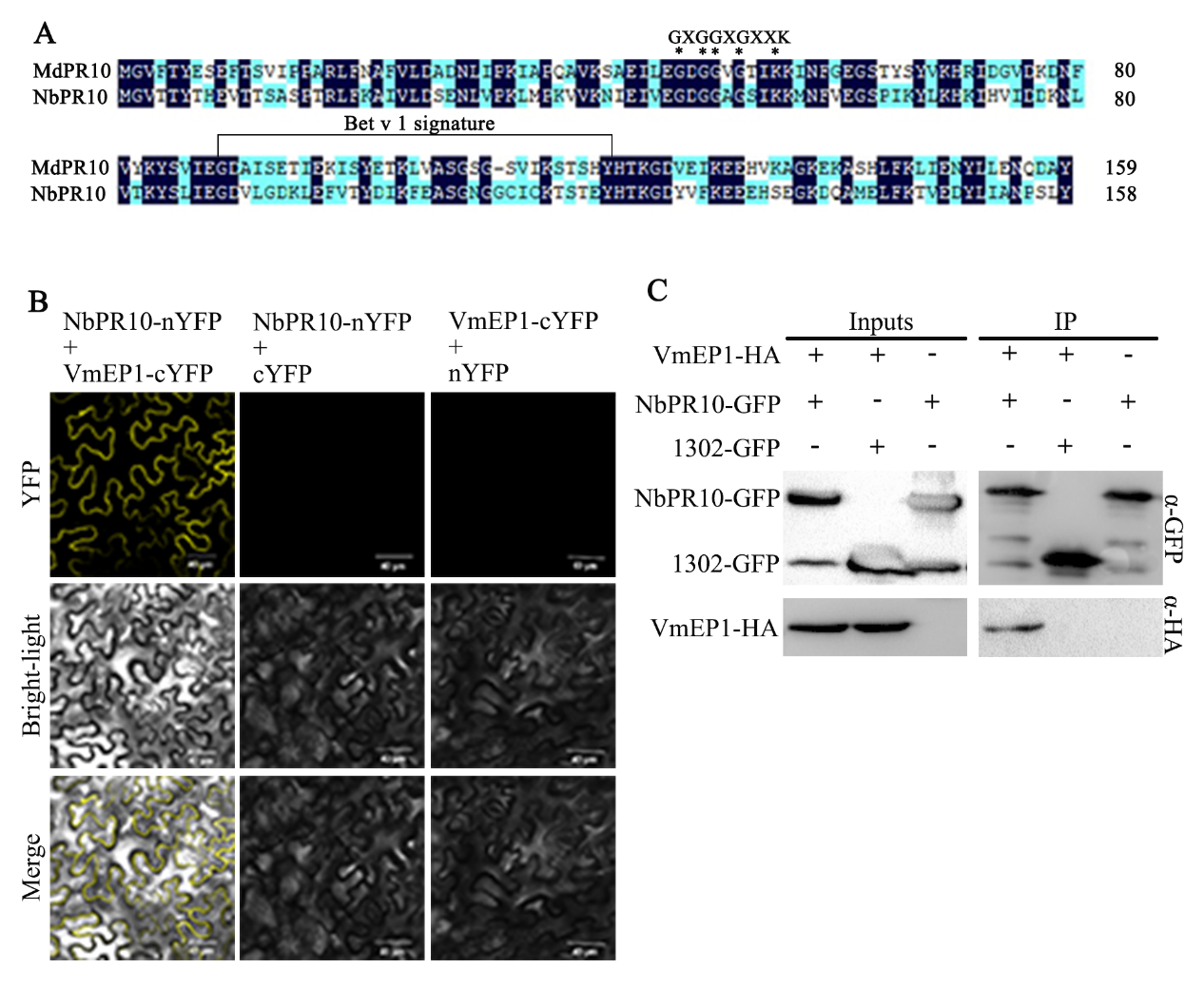


**Supplementary Figure 2.** (A) Alignment of amino acid sequences of MdPR10 and NbPR10. The alignment was constructed by using ClustalW in MEGA7 software. The picture was exported by DNAMAN. (B) Bimolecular fluorescence complementation showed that VmEP1 interacted with NbPR10 in leaf cells of *N. benthamiana*. VmEP1-cYFP and NbPR10-nYFP were co-expressed in *N. benthamiana* by agro-infiltration. The yellow fluorescence was observed 48–72 h post infiltration (Bars = 20 µm). (C) In vivo Co-IP assay of HA: VmEP1 (without the signal peptide sequence) and GFP-NbPR10. Both genes were expressed in *N. benthamiana* leaves using agro-infection. The input experiment was performed by western blot with the HA antibody and GFP antibody to confirm the expression of the two proteins. The mixed proteins were blended with GFP-trap agarose beads. The final eluent was analyzed by immunoblot using above-mentioned antibodies to detect VmEP1 and MdPR10. This assay was repeated three times.


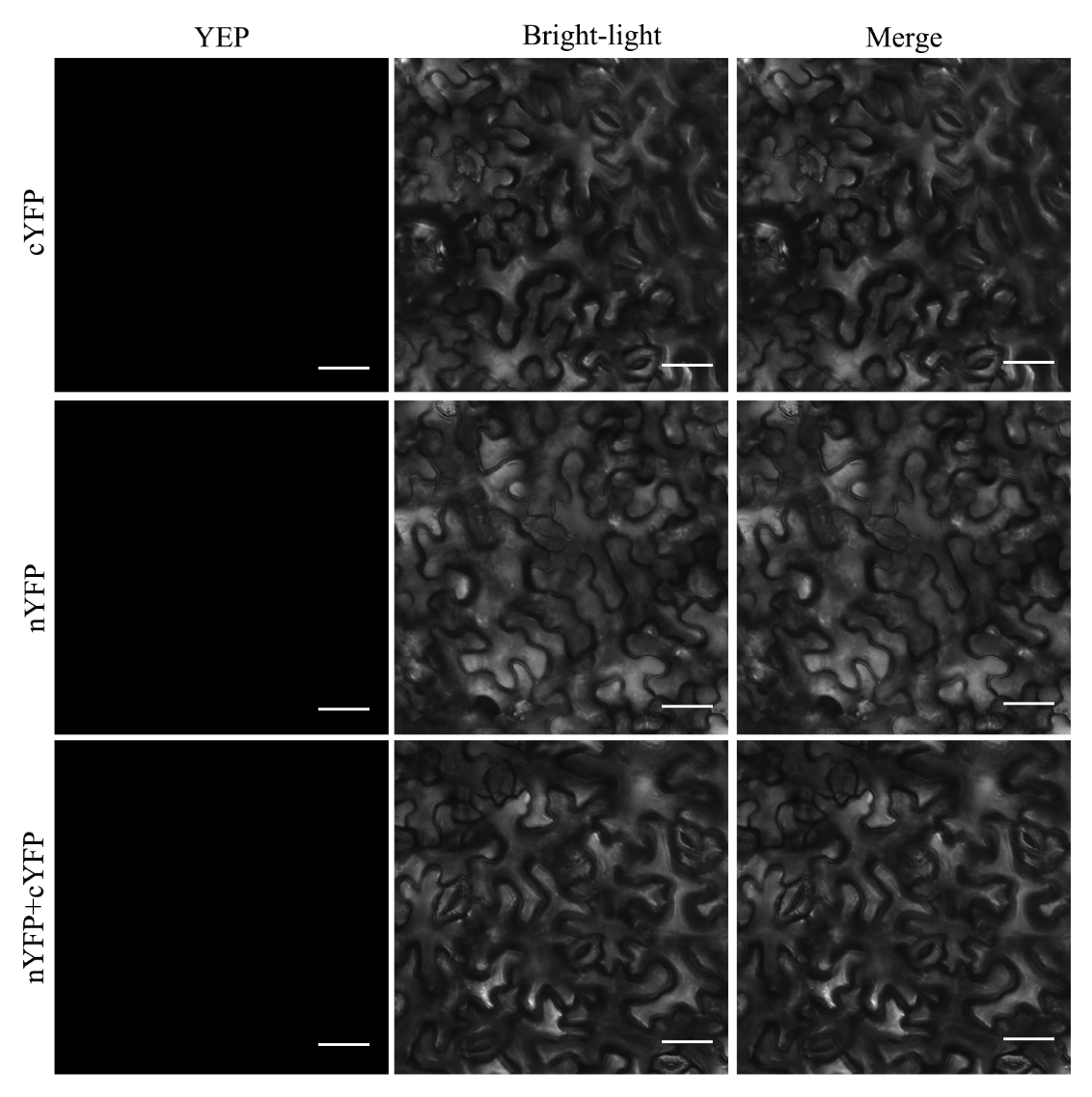


**Supplementary Figure 3.** Bimolecular fluorescence complementation showed that cYFP did not interact with nYFP in leaf cells of *N. benthamiana*. cYFP and nYFP were co-expressed in *N. benthamiana* by agro-infiltration. The yellow fluorescence was observed 48–72 h post infiltration (Bars = 20 µm).


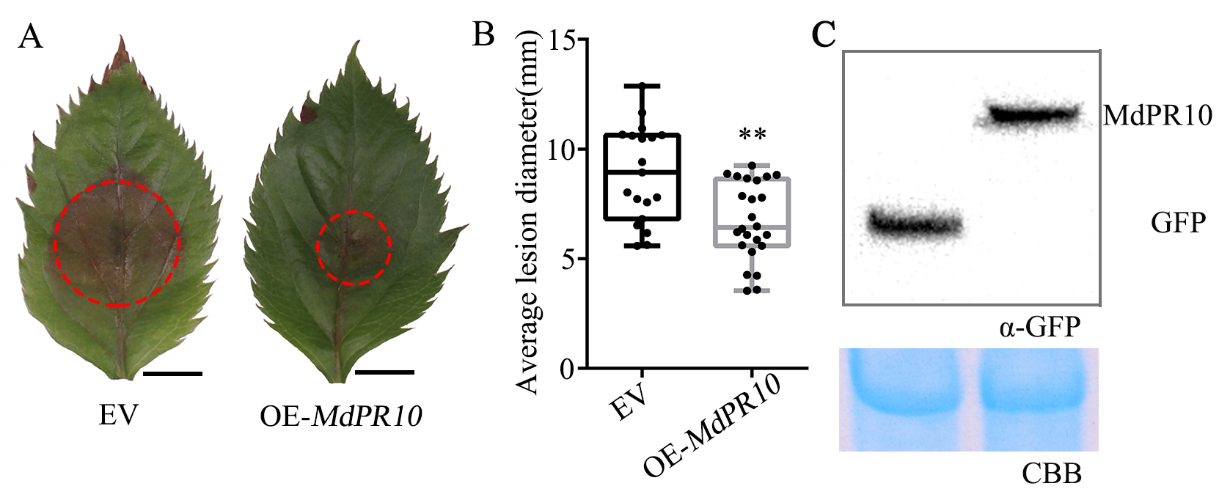


**Supplementary Figure 4.** Transient expression of *MdPR10* in apple enhances resistance to *C. gloeosporioides*. (A) Representative disease symptoms of apple leaves transient expressing *MdPR10* at 36 h after inoculation of *C. gloeosporioides.* Bars=5mm. (B) The average lesion semidiameter in apple leaves in which MdPR10 is transiently expressed was evaluated at 48 h after inoculation of *C. gloeosporioides*. Error bars represent standard deviations calculated from three biological replicates, each performed with at least 20 apple leaves. The statistical analyses were performed with Student’s t test: **P<0.01. (C) Immunoblot analysis of proteins in apple transiently expressing green fluorescent protein (GFP) control and MdPR10 fused with GFP tag.


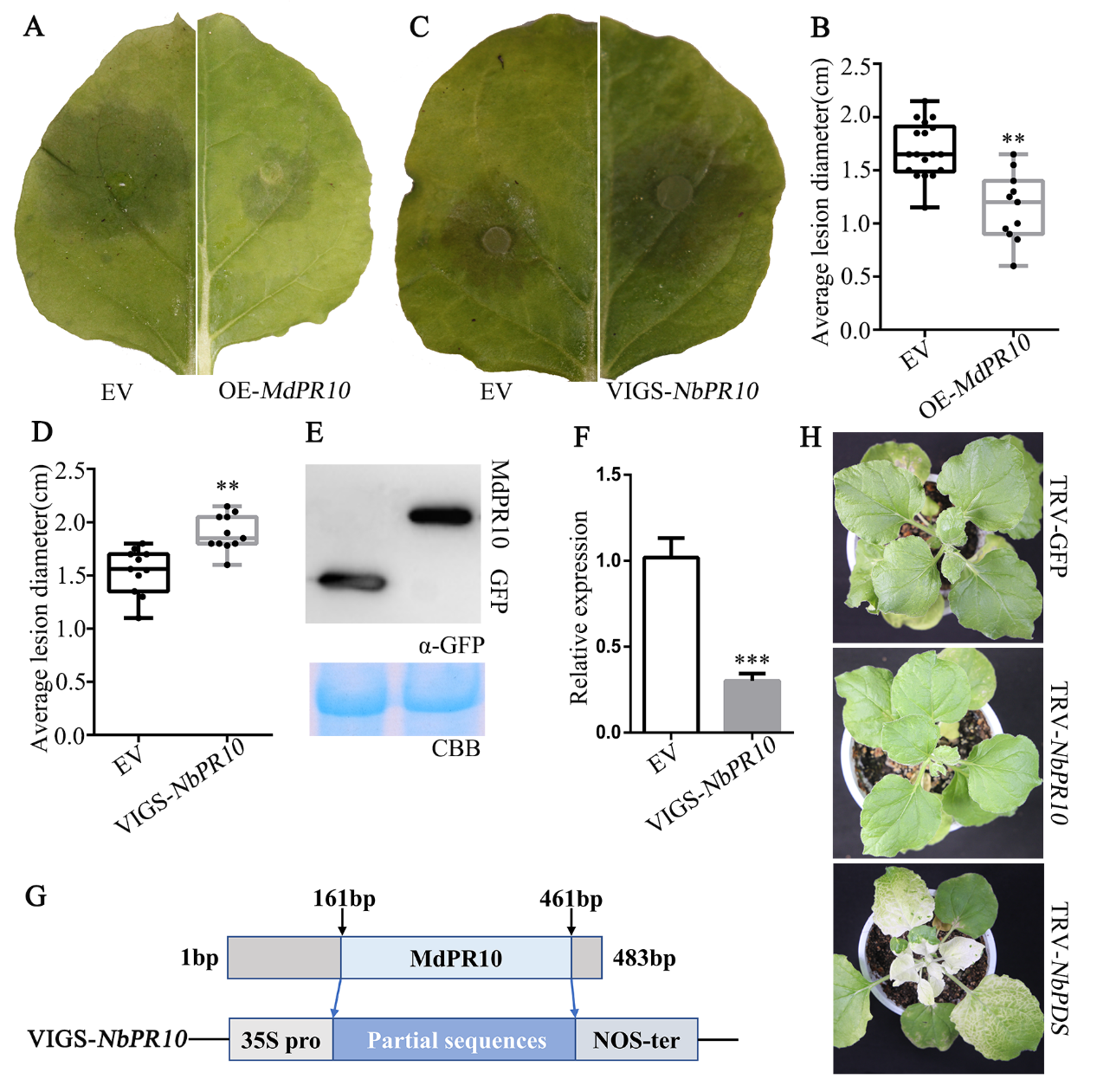


**Supplementary Figure 5.** PR10 positively regulates plant resistance to the oomycete *P. caosici* in *N. benthamiana.* (A) Representative disease symptoms of *N. benthamiana* leaves transient expressing *MdPR10* at 48 h after inoculation of *P. capsica.* The leaves (n>10) were inoculated with *P. capsica* fresh mycelial plugs and photographed 36 h post-inoculation (hpi). The experiment was repeated three times with similar results. (B) The average lesion diameter in *N. benthamiana* leaves overexpressing *MdPR10* was evaluated at 36 h after inoculation of *P. capsica*. (C) Representative disease symptoms of *N. benthamiana* leaves silencing *NbPR10* at 36 h after inoculation of *P. capsica.* The leaves (n>10) were inoculated with *P. capsica* fresh mycelial plugs and photographed 36 h post-inoculation (hpi). The experiment was repeated three times with similar results. (D) The average lesion semidiameter in *N. benthamiana* leaves silencing *NbPR10* was evaluated at 36 h after inoculation of *P. capsica*. (E) Immunoblot analysis of proteins in apple transiently expressing green fluorescent protein (GFP) control and MdPR10 fused with GFP tag.

(F) Silence efficiency detection of *NbPR10* in *N. benthamiana* by RT-PCR 3 weeks after infiltration of VIGS-*NbPR10*. The transcripts levels of genes were normalized to the actin gene of *N. benthamiana*. (G) Schematic of constructs used to silence *NbPR10*. (H) Representative images of the TRV-*NbPR10* VIGS plants compared to the TRV-GFP control showing that there were no obvious developmental phenotypes observed in these plants. In (B),(D) and (F):Error bars represent standard deviations calculated from three replicates. The statistical analyses were performed with Student’s t test: ***P<0.001, **P<0.01.


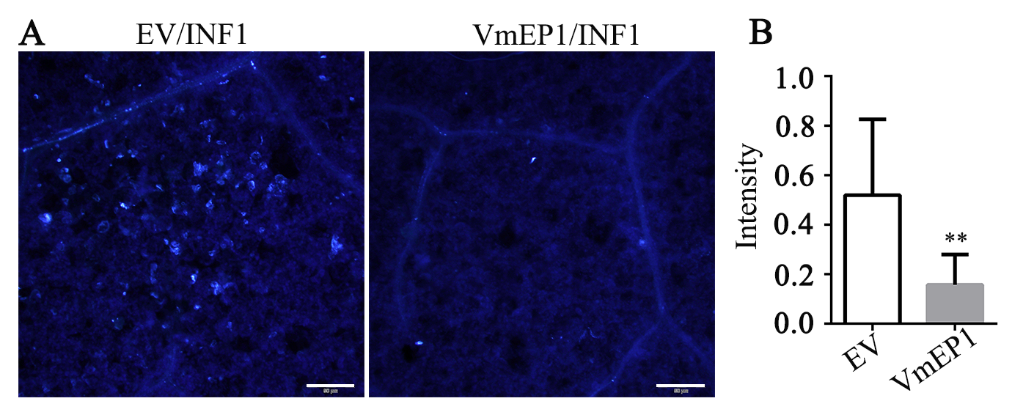


**Supplementary Figure 6.** VmEP1 compromises INF1-induced cllose accumulation in *N. benthamiana.* (A) Trypan blue staining with leaves at 48 h after co-expression of VmEP1 and INF1 in *N. benthamiana*. Co-expression of EV and INF1 as control. Bars = 50 μm. (B) Quantification of callose deposition in leaf tissues, as determined by ImageJ software. Error bars represent standard deviations calculated from three biological replicates, each performed with at least six technical replicates. The statistical analyses were performed with Student’s t test: **P<0.01.
