# Supplementary material for: A Valsa mali Effector Protein 1 Targets Apple (Malus domestica) Pathogenesis-Related 10 Protein to Promote Virulence
Source: Front Plant Sci. 2021 Oct 7;12:741342. doi: 10.3389/fpls.2021.741342 (PMC8528966; doi:10.3389/fpls.2021.741342)
Supplement: Supplementary file 2 [file Table_1.docx]

Table 1 Primers used in this study

| **Primer name** | **Sequence** | **Purpose** |
| --- | --- | --- |
| TRV2:NbPR10-F | GTGAGCTCGGTACCggatcc CAAGAAGATGAACTTTGTG | Clone NbPR10 to TRV2 for expression in *N. benthamiana* |
| TRV2:NbPR10-R | GAGTAAGGTTACCgaattc GCAAGAAGGTAGTCTTCAAC |  |
| 1302-MdPR10-GFP-F | GGGGACTCTTGACCatggta ATGGGTGTTTTCACATACGAATC | Clone MdPR10 to pCAMBIA1302 for expression in *N. benthamiana* |
| 1302-MdPR10-GFP-R | CTCACCATCCTAGGactagt GTTGTAGGCATCCTGATTCTCC |  |
| 1302-NbPR10-GFP-F | GGGGACTCTTGACCatggta ATGGGTGTCACTACCTATACTC | Clone NbPR10 to pCAMBIA1302 for expression in *N. benthamiana* |
| 1302-NbPR10-GFP-R | CTCACCATCCTAGGactagt AACATAGAGAGAAGGATTAGCG |  |
| 1302-VmEP1-GFP-F | GGGGACTCTTGACCatggta ATGAGCCTTGTTACTACCTGCG | Clone VmEP1 to pCAMBIA1302 for expression in *N. benthamiana* |
| 1302-VmEP1-GFP-R | CTCACCATCCTAGGactagt TCAGTCTACCGAACATGTCTGTGG |  |
| nYFP-MdPR10-F | GCCACAACATCGAGggatcc ATGGGTGTTTTCACATACGAATC | Clone MdPR10 to nYFP for expression in *N. benthamiana* |
| nYFP-MdPR10-R | TTCGAGCTCTATcccggg GTTGTAGGCATCCTGATTCTCC |  |
| nYFP-NbPR10-F | GCCACAACATCGAGggatcc ATGGGTGTCACTACCTATACTC | Clone NbPR10 to nYFP for expression in *N. benthamiana* |
| nYFP-NbPR10-R | TTCGAGCTCTATcccggg AACATAGAGAGAAGGATTAGCG |  |
| cYFP-VmEP1-F | GCCCAAGCTTCGACtctaga ATGAGCCTTGTTACTACCTGCG | Clone VmEP1 to cYFP for expression in *N. benthamiana* |
| cYFP-VmEP1-R | ACGCTGCCGTCCATggatcc TCAGTCTACCGAACATGTCTGTGG |  |
| PCH-VmEP1-F | TTACAATTATCGATACAATG TACCCATACGACGTCCCAGACTACGCT ATGAGCCTTGTTACTACCTGCG | Clone VmEP1 to PCH for expression in *N. benthamiana* |
| PCH-VmEP1-R | CTCATTAAAGCAGGACAAGC TCAGTCTACCGAACATGTCTGTGG |  |
| qNbPR10-F | CCCCAACTAGATTATTCAAAGC | qRT-PCR analysis in *N. benthamiana* |
| qNbPR10-R | GACCACCTTCCACAAAGTTCAT |  |
| qMdPR10-F | ATGCTGATAACCTCATCCCCAA | qRT-PCR analysis in *M. domestica* |
| qMdPR10-R | GCCTTCACCAAAAGTGATCTTCTT |  |
| qMdCalS5-F | AAGAATCTATGTCGGGAGG |  |
| qMdCalS5-R | TGATAATCTACACGACGAATG |  |
| qMdEF1α-F | \| ATTCAAGTATGCCTGGGTGC \| \| --- \| |  |
| qMdEF1α-R | CAGTCAGCCTGTGATGTTCC |  |
| qVmEP1-F | TTGACAACAACCACGGTTACGA | qRT-PCR analysis in *M. domestica* and *N. benthamiana* |
| qVmEP1-R | TGCTGACGCATACCCTCCAT |  |
| qVmG6PDH-F | GCTGTGCTCGCTCGTTGCTT |  |
| qVmG6PDH-F | AACTGCTGCTTGTCGCCCTC |  |
